# Microtubule‐Associated Protein Tau (MAPT) in non‐transgenic and transgenic P301S mouse lenses

**DOI:** 10.1002/alz.089539

**Published:** 2025-01-03

**Authors:** Juliet A Moncaster, Olga Minaeva, Lee E Goldstein

**Affiliations:** ^1^ Boston University Alzheimer’s Disease Research Center, Boston, MA USA; ^2^ Boston University Chobanian & Avedisian School of Medicine, Boston, MA USA; ^3^ Boston University Alzheimer's Disease Research Center, Boston, MA USA

## Abstract

**Background:**

We previously discovered that Aβ accumulates in the cortical/supranuclear region of the lens in people with Alzheimer’s Disease (AD) (Goldstein et al., 2003) and Down Syndrome (DS; (Moncaster et al., 2010). We also demonstrated Aβ in the Tg2576 APP Swedish mutation AD mouse model (Moncaster et al., 2022). Another protein that is involved in AD is Microtubule‐Associated Protein Tau (MAPT). Tau is expressed in the brain and becomes hyperphosphorylated in AD eventually forming neurofibrillary tangles. Tau has previously been reported to be expressed in the mouse lens (Bai et al., 2007; Zhao et al., 2013) but the results vary depending on the mouse model and antibodies used. Here we investigated whether tau protein was expressed in non‐transgenic mouse lenses and in a transgenic tau mutant (P301S) and whether there was a cataract phenotype.

**Method:**

P301S tau mutant transgenic and non‐transgenic mice were bred and maintained at Boston University. Breeder mice were purchased from The Jackson Laboratory, Bar Harbor, ME. Male and Female transgenic and non‐transgenic mice were sacrificed throughout their lifespan at ages 3‐12 months. Mice were perfused with phosphate buffered saline, lenses were isolated and then imaged under two different sources of light using a Nikon camera and a custom‐adapted Zeiss stereophotomicroscope. Lenses were then snap frozen and analyzed by Western using a panel of tau antibodies.

**Result:**

P301S transgenic and non‐transgenic mouse lenses all expressed tau during their lifespan when analyzed by Western blot. P301S transgenic lenses demonstrated an additional higher molecular weight band compared to the non‐transgenic mice. No cataract phenotype was observed in transgenic or non‐transgenic mouse lenses.

**Conclusion:**

There was no overt difference in lens phenotype between the P301S transgenic and non‐transgenic mice. However, the banding pattern observed by Western blot for P301S transgenic mice compared to non‐transgenic was different. Our data suggest the P301S tau mutation affects tau processing but does not result in a cataract phenotype. Based on our current and previous results, the data suggests that Aβ may play a more significant role than tau in lens pathology in AD.

